# Development and validation of robust metabolism‐related gene signature in the prognostic prediction of hepatocellular carcinoma

**DOI:** 10.1111/jcmm.17718

**Published:** 2023-03-15

**Authors:** Yangxun Pan, Deyao Zhang, Yuheng Chen, Huake Li, Jiongliang Wang, Ze Yuan, Liyang Sun, Zhongguo Zhou, Minshan Chen, Yaojun Zhang, Dandan Hu

**Affiliations:** ^1^ Department of Liver Surgery, State Key Laboratory of Oncology in South China Sun Yat‐sen University Cancer Center, Sun Yat‐sen University Guangzhou China; ^2^ Hepatobiliary Center The First Affiliated Hospital of Nanjing Medical University & Research Unit of Liver Transplantation and Transplant Immunology, Chinese Academy of Medical Sciences Nanjing China; ^3^ Department of Oncology Changning County People's Hospital Baoshan China

**Keywords:** hepatocellular carcnioma, metabolic studies, nomogram, prognosis

## Abstract

Hepatocellular carcinoma (HCC) is one of the most common malignant tumours worldwide. Given metabolic reprogramming in tumours was a crucial hallmark, several studies have demonstrated its value in the diagnostics and surveillance of malignant tumours. The present study aimed to identify a cluster of metabolism‐related genes to construct a prediction model for the prognosis of HCC. Multiple cohorts of HCC cases (466 cases) from public datasets were included in the present analysis. (GEO cohort) After identifying a list of metabolism‐related genes associated with prognosis, a risk score based on metabolism‐related genes was formulated via the LASSO‐Cox and LASSO‐pcvl algorithms. According to the risk score, patients were stratified into low‐ and high‐risk groups, and further analysis and validation were accordingly conducted. The results revealed that high‐risk patients had a significantly worse 5‐year overall survival (OS) than low‐risk patients in the GEO cohort. (30.0% vs. 57.8%; hazard ratio [HR], 0.411; 95% confidence interval [95% CI], 0.302–0.651; *p* < 0.001) This observation was confirmed in the external TCGA‐LIHC cohort. (34.5% vs. 54.4%; HR 0.452; 95% CI, 0.299–0.681; *p* < 0.001) To promote the predictive ability of the model, risk score, age, gender and tumour stage were integrated into a nomogram. According to the results of receiver operating characteristic curves and decision curves analysis, the nomogram score possessed a superior predictive ability than conventional factors, which indicate that the risk score combined with clinicopathological features was able to achieve a robust prediction for OS and improve the individualized clinical decision making of HCC patients. In conclusion, the metabolic genes related to OS were identified and developed a metabolism‐based predictive model for HCC. Through a series of bioinformatics and statistical analyses, the predictive ability of the model was approved.

## 1 INTRODUCTION

The incidence and mortality rates of primary liver cancer remain the sixth most common and third leading cause of cancer deaths worldwide in 2020, respectively, among which hepatocellular carcinoma (HCC) comprised 75%–85% of the reported pathologic type of primary liver cancer.^1^ Since HCC generally progresses asymptomatically, most patients are diagnosed with intermediate or even advanced stages of which the radical therapies are impossible to be performed, and their long‐term prognosis are poor.^2^ According to the American Joint Committee on Cancer (AJCC) tumour‐node‐metastasis (TNM) staging system, liver transplantation, hepatectomy, interventional therapy and systemic therapy have achieved a variety of therapeutic effects for patients diagnosed with corresponding stages and prolonged survival periods.^3^ However, several patients with a similar tumour stage finally obtain dramatically different clinical outcomes, indicating that the TNM staging system is still need to be improved. Those high‐risk HCC patients with potentially poor outcomes must be monitored and recommended to timely and effective treatments to prolong prognosis and improve qualities of life.^4^ Therefore, there is an urgent need for effective prediction biomarkers to accurately assess the prognosis of HCC patients.

Additionally, alpha‐fetoprotein (AFP), des‐gamma‐carboxy prothrombin (DCP) and carbohydrate antigen (CA) 19–9 demonstrated promising values in clinical prediction for HCC patients prognosis who underwent resection or transcatheter arterial chemoembolization.^5^, ^6^ Presently, with the developments in gene chips and high‐throughput sequencing, gene biomarkers including microRNA, circular RNA and mRNA were proven their increasing importance in terms of the application for prognosis of HCC.^7^ Moreover, recently, there were many pieces of studies developing prognostic stratifications that were able to classify HCC patients into different risk groups based on the multigene expressions.^8^, ^9^, ^10^ Unfortunately, these studies were limited by the sample size, mainly based on the cancer genome atlas (TCGA) dataset and failed to perform an external validation to estimate the possibility for optimism and overfitting in model performance.^11^

On the other hand, cellular metabolic reprogramming is a critical hallmark during carcinogenic progression and is dramatically associated with the tumorigenesis.^12^, ^13^ As early as 1920s, Warburg et al. observed that an increment of glucose was consumed by tumour tissues compared to normal tissues.^14^ In addition, excessive activated anaerobic glycolysis, as well as insufficient aerobic respiration, were regarded as the specific features of tumour cells,^15^ which were also observed in HCC tissues.^16^ Apart from the metabolism of glucose, lipid, amino acid, nucleotide, and other metabolite metabolism also demonstrated varying degrees of alternations in the procedure of HCC.^17^, ^18^ With the increasing applications of bioinformatics analysis in the diagnostic and prognostic predictions of carcinoma, several researchers have associated the metabolome with the genome, which allowed broad and precise metabolite profiles to be depicted.^19^, ^20^

Accordingly, it is necessary to take metabolomics, genomics and transcriptomics into comprehensive consideration, which would help to better understand the biological behaviour of malignant tumours. Previous studies have initially proven the utility for metabolism‐related genes to generate a predictive model for colorectal cancer, bladder cancer and breast cancer and achieved promisingly predictive abilities.^21^, ^22^, ^23^ Whereas, there is little research using the transcriptomics profiles of metabolism‐related genes to evaluate the outcomes in predicting HCC patients. Therefore, the present study aims to identify a group of the metabolism‐related genetic cluster to construct a predictive model for HCC patients.

## 2 METHODS AND MATERIALS

### 2.1 Collection of data

The gene expression profiling of HCC was downloaded from the Gene Expression Omnibus (GEO; https://www.ncbi.nlm.nih.gov/geo/) database. The detailed inclusion criteria for candidate datasets were following: Human gene expression profile; hepatocellular carcionoma specimen; total count of sample volume ≥60; availability of follow‐up information (overall survival, [OS]) and related clinical data. Finally, a total of four datasets (GSE14520, GSE54236, GSE10141 and GSE116174) were included in the present study (GEO cohort).

For the purpose of estimating the power and robustness of the model, the cancer genome atlas hepatocellular carcinoma (TCGA‐LIHC) cohort, as the external validation cohort (TCGA‐LIHC cohort), was obtained from the University of California, Santa Cruz (UCSC) Xena website (https://gdc.xenahubs.net). Furthermore, to evaluate the specificity of the metabolic gene signature derived from HCC, the mRNA sequencing data of the remaining 32 TCGA tumours were downloaded from UCSC Xena website.

In the present study, availably clinical parameters including age, gender, tumour stage based on American Joint Committee on Cancer (AJCC), AFP levels, ethnicity, survival status and survival time were collected. Subsequently, the normal tissues adjacent to cancer, intrahepatic cholangiocarcinoma and cases that lacked survival information were excluded from these datasets.

### 2.2 Data processing

The mRNA microarray datasets from the GEO database were normalized before being downloaded. Probe identifications of gene matrix files were transformed into gene symbols according to the annotation file from the corresponding platform. The sequencing data were normalized to the Fragments Per Kilobase Million (FPKM) value. All gene expression values were processed by log2, moreover the average value was calculated as the final expression value if multiple probes corresponded to the same gene symbol. Given there was a need for a combination of multiple datasets, the Empirical Bayes method by ‘sva’ package was applied to diminish the batch effects among datasets after merging.^24^ Additionally, the same method was applied to correct the batch effect due to different omics platforms between GEO cohort and TCGA‐LIHC cohort before external validation. (Figure S1).

### 2.3 Extraction of metabolism‐related genes

In the current study, the whole metabolism‐related genes were derived from Kyoto Encyclopaedia of Genes and Genomes (KEGG) metabolism‐related genesets of ‘c2.cp.kegg.v7.0.symbols.gmt’ (http://software.broadinstitute.org/gsea/downloads.jsp/). After intersecting the whole geneset of samples with the metabolic gene sets, 482 metabolism‐related genes were identified in the transcriptome data. The expression levels of these genes was extracted from each sample to undergo further analysis.

### 2.4 Construction and validation of the metabolism‐related signature

In the GEO cohort, univariate Cox regression analysis was performed via ‘survival’ package to screen out the metabolic genes that were correlated with OS (*p* < 0.005). To address the impact of overfitting, the Least Absolute Shrinkage and Selection Operator (LASSO) algorithm was performed to select potential genes to construct metabolic gene signature by using ‘glmnet’ and ‘biospear’ packages.^25^, ^26^

The values of penalty parameter λ were determined by 100‐fold cross‐validations. In order to maximally reduce the false discover rate (FDR), the methods of cross‐validated log‐likelihood (LASSO‐Cox) and penalty cross‐validated likelihood (LASSO‐pcvl) were applied, and the final biomarkers were determined by the common parts of both methods. Finally, the common biomarkers filtered out by LASSO‐Cox and LASSO‐pcvl algorithms were used to develop a formula comprising the gene expression levels (Expr) weighted by the corresponding coefficients:

$$\text{Risk score}=\left[\text{Expr of gene}\;\left(1\right)\times \text{coefficient of gene}\;\left(1\right)\right]+\left[\text{Expr of gene}\;\left(2\right)\times \text{coefficient of gene}\;\left(2\right)\right]+\ldots +\left[\text{Expr of gene}\;\left(n\right)\times \text{coefficient of gene}\left(n\right)\right].$$

According to the optimal cut‐off value of the GEO cohort calculated via ‘survminer’ package, GEO cohort was stratified into high‐ and low‐risk groups, respectively. Subsequently, the multivariate Cox regression analyses were applied to determine whether the risk score was an independent prognostic factor for HCC. Additionally, calibration curves, receiver operating characteristic (ROC) analysis and decision curve analysis (DCA) were employed to evaluate the accuracy and clinical utility of the model for OS via the ‘ROCR’ and ‘rms’ packages.^27^

### 2.5 Bioinformatic analysis

Based on Hallmarks geneset of ‘h.all.v7.0.symbols.gmt’, Gene set enrichment analysis (GSEA) was applied to identify the significantly enriched pathways between the high‐ and low‐risk groups via the ‘DESeq2’ package. Metascape tool (http://metascape. org/) was carried out to explore the functional annotations of the metabolism‐related genes selected by the LASSO‐Cox and LASSO‐pcvl algorithms.^28^ Nevertheless, the associations between the risk score and infiltrated immune cells were investigated using CIBERSORTx and gene‐centric single sample GSEA (ssGSEA) algorithm via the ‘IOBR’ and ‘GSVA’ packages, respectively. The CIBERSORTx algorithm was able to identify 22 types of human immune cell phenotypes according to the gene expression data, while, the ssGSEA algorithm was applied to further explore the phenotypes of the immune cell subsets.^29^, ^30^

### 2.6 Statistical analysis

All statistical analyses were performed via using R software version 4.2.2 (http://www.r‐project.org). Categorical variables were compared by chi‐square test, and continuous variables were evaluated with the Wilcoxon and Kruskal–Wallis tests. OS was defined as the length of time from the date of diagnosis to death from any cause. The survival curves between the two groups were estimated using the Kaplan–Meier method (K‐M curves), and significant differences were validated by using the log‐rank test. All *p* values were based on two‐sided statistical tests, and *p* < 0.050 was considered statistically significant unless specified.

## 3 RESULTS

### 3.1 Patient characteristics and the construction of the risk score

**FIGURE 1:**
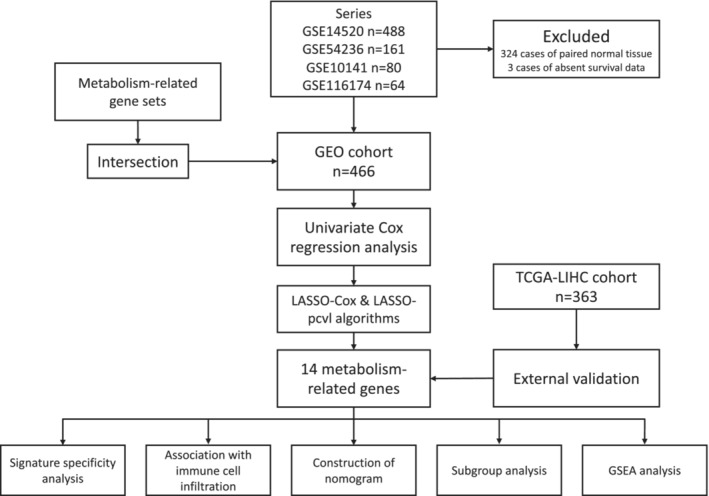
Flow diagram of construction and validation for the metabolism‐related gene signature model. LASSO, least absolute shrinkage and selection operator; GSEA, gene set enrichment analysis.

**FIGURE 2:**
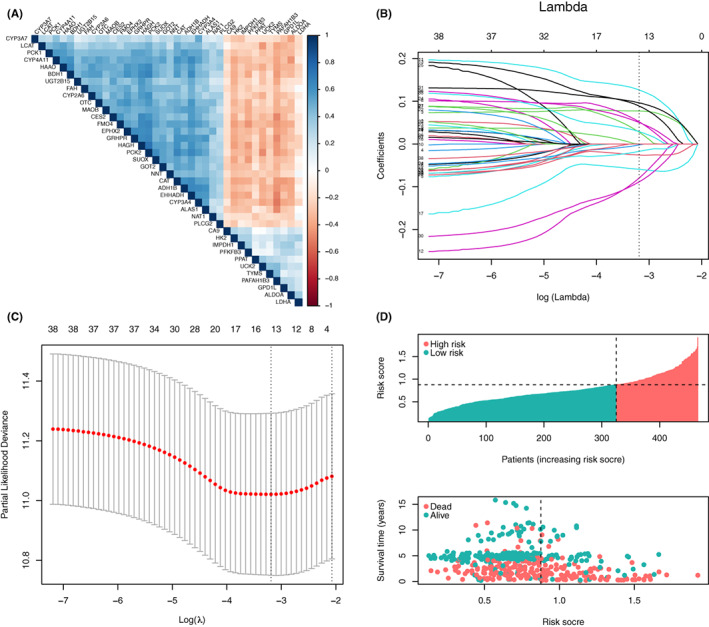
Construction of the risk score in the GEO cohort. (A) The correlation analysis of the 36 metabolic genes. (B) The LASSO‐Cox coefficient of the 14 metabolic genes. The dotted vertical line indicated the value of lambda selected by 100‐fold cross‐validation via minimum criteria. (C) The 100‐fold cross‐validation for variable selection in the LASSO‐Cox model. The two dotted vertical lines indicate the optimal values by using the minimum criteria and the 1‐SE criteria, respectively. (D) Distribution of the risk score and survival state of patients in the GEO cohort. LASSO, least absolute shrinkage and selection operator; GEO, gene expression omnibus; SE, standard error.

According to the Figure 1, 324 cases of normal tissue adjacent to cancer, three cases that lacked survival information were excluded initially from the enrolled four datasets. Finally, a total of 466 cases HCC tissue were included in the followed analysis, and the characteristics of included patients were demonstrated in Table 1. In the GEO cohort, the univariate Cox regression analysis was conducted to screen out metabolic genes related to OS. As a result, 38 metabolic genes (*p* < 0.005) were filtered out to achieve further analysis. Moreover, strong correlations among these genes were observed in the GEO cohort. (Figure 2A) Hence, we introduced these genes to the LASSO‐Cox and LASSO‐pcvl algorithms to diminish the overfitting and construct the model.

**TABLE 1:** Clinicopathological characteristics.

		GEO cohort (*N* = 305)^a^		TCGA‐LIHC cohort (*N* = 363)	
Variable		High risk	Low risk	High risk	Low risk
N		98	207	107	256
Risk score, median [IQR]		1.13 [0.98, 1.30]	0.59 [0.44, 0.72]	1.12 [1.02, 1.36]	0.57 [0.45, 0.70]
Gender	Female	7 (7.14)	30 (14.49)	46 (42.99)	72 (28.12)
	Male	91 (92.86)	177 (85.51)	61 (57.01)	184 (71.88)
Age	<60 years	82 (83.67)	160 (77.29)	60 (56.07)	113 (44.14)
	≥60 years	16 (16.33)	47 (22.71)	47 (43.93)	143 (55.86)
Tumour stage	I/II	55 (56.12)	172 (83.09)	57 (53.27)	197 (76.95)
	III/IV	34 (34.69)	27 (13.04)	43 (40.19)	42 (16.41)
	Unknown	9 (9.18)	8 (3.86)	7 (6.54)	17 (6.64)
Hepatitis B virus	Negative	19 (19.39)	22 (10.63)	NR	NR
	Positive	79 (80.61)	185 (89.37)	NR	NR
Alpha‐fetoprotein	≥300 ng/mL	49 (63.64)	60 (36.59)	NR	NR
	<300 ng/mL	26 (33.77)	102 (62.20)	NR	NR
	Unknown	2 (2.60)	2 (1.22)	NR	NR
Ethnicity	American Indian or Alaska native	NR	NR	0 (0.00)	1 (0.39)
	Asian	NR	NR	54 (50.47)	101 (39.45)
	Black or African American	NR	NR	4 (3.74)	13 (5.08)
	White	NR	NR	49 (45.79)	131 (51.17)
	Unknown	NR	NR	0 (0.00)	10 (3.91)
Prior malignancy	No	NR	NR	102 (95.33)	227 (88.67)
	Yes	NR	NR	5 (4.67)	29 (11.33)
Treatment types	Pharmaceutical therapy	NR	NR	53 (49.53)	121 (47.27)
	Radiation therapy	NR	NR	54 (50.47)	135 (52.73)
Overall survival	Alive	37 (37.76)	146 (70.53)	56 (52.34)	177 (69.14)
	Dead	61 (62.24)	61 (29.47)	51 (47.66)	79 (30.86)
Survival time, year, median [IQR]		1.92 [0.79, 4.07]	4.47 [2.85, 4.94]	1.12 [0.56, 2.31]	1.77 [1.08, 3.42]

Abbreviations: GEO, gene expression omnibus; IQR, interquartile range; NR, not reported; TCGA‐LIHC, the cancer genome atlas liver hepatocellular carcinoma.

^a^ Clinicopathological characteristic data that was available from the GEO datasets was described here.

After performing the LASSO‐Cox and LASSO‐pcvl algorithms, a total of 14 overlapped metabolism‐related biomarkers were induced to develop the risk score formula (Figure 2B,C):

Risk score = HK2 × (0.0771) + TYMS × (0.0503) + UCK2 × (0.1263) + NAT1 × (−0.0797) + GPD1L × (0.0888) + CYP4A11 × (−0.0258) + CYP3A7 × (−0.0022) + HAGH × (−0.0593) + LDHA × (0.0276) + SUOX × (−0.0172) + PLCG2 × (−0.0886) + CA9 × (0.0963) + PFKFB3 × (0.0289) + PPAT × (0.0519).

Subsequently, the risk scores for each case were calculated based on the formula mentioned above, and the optimal cut‐off value of 0.880 was determined, which was able to generate the largest survival difference between the high‐ and low‐risk groups. (Figure S2) In the GEO cohort, the distribution of risk scores and the survival status of patients were displayed. (Figure 2D).

### 3.2 Validation and evaluation of the metabolic gene signature

**FIGURE 3:**
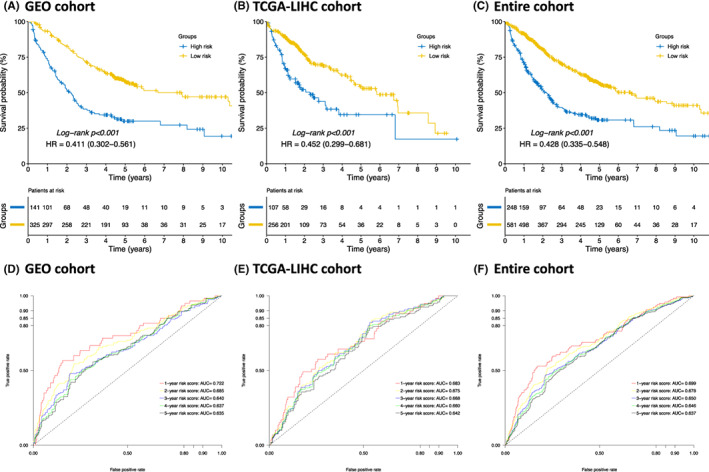
The association between the overall survival and risk score. (A–C) The Kaplan–Meier curves for overall survival between the high‐ and low‐risk groups in terms of the GEO, TCGA‐LIHC and entire cohorts, respectively. (D–F) The time‐dependent ROC curves for the risk score in the GEO, TCGA‐LIHC and entire cohorts, respectively. GEO, gene expression omnibus; TCGA‐LIHC, the cancer genome atlas of liver hepatocellular carcinoma; ROC, receive operating characteristic; HR, hazard ratio; AUC, area under curve.

According to the result from Figure 3A, high‐risk patients remarkably suffered from a worse 5‐year OS compared to low‐risk patients in the GEO cohort (30.0% vs. 57.8%; hazard ratio [HR], 0.411; 95% confidence interval [95% CI], 0.302–0.651; *p* < 0.001). In additional, the multivariate results of Cox regression analysis revealed that classified as low‐risk score (HR, 0.374; 95% CI, 0.256–0.547; *p* < 0.001) as well as I/II tumour stages (HR, 0.492; 95% CI, 0.323–0.748; *p* < 0.001) were significantly associated with preferable OS. (Table 2).

**TABLE 2:** Univariate and multivariate Cox regression analyses of the risk score and clinical characteristics with the overall survival in the GEO cohort.

	Univariable analysis		*p*‐Value	Multivariable analysis		
Variable	HR	95% CI		HR	95% CI	*p*‐Value
Age (≥60 years vs. <60 years)	0.888	0.568–1.388	0.603			
Gender (male vs. female)	2.024	1.026–3.992	**0.042**			
Tumour stage (I/II vs. III/IV)	0.363	0.187–0.703	**0.003**	0.492	0.323–0.748	**<0.001**
Risk score (low risk vs. high risk)	0.304	0.213–0.435	**<0.001**	0.374	0.256–0.547	**<0.001**

Abbreviations: 95% CI, 95% confidence interval; GEO, gene expression omnibus; HR, hazard ratio.

To further externally validate the prognostic value of the signature in TCGA‐LIHC cohort, the same formula was applied to the TCGA‐LIHC cohort for calculating the risk scores for each case. Similarly, patients in the TCGA‐LIHC cohort were divided into high‐ and low‐risk groups based on the same cut‐off value of 0.880 as the GEO cohort. The results indicated that significantly different 5‐year OS rates were shown between the high‐ and low‐risk groups in terms of the TCGA‐LIHC cohort. (34.5% vs. 54.4%; HR 0.452; 95% CI, 0.299–0.681; *p* < 0.001; Figure 3B) This finding was consistent with observations from the GEO cohort. Accordingly, multivariate Cox regression analysis revealed that classified as low‐risk score (HR, 0. 522; 95% CI, 0.358–0.762; *p* < 0.001) as well as I/II tumour stages (HR, 0.480; 95% CI, 0.325–0.708; *p* < 0.001) were independently protective factors for OS in the TCGA‐LIHC cohort. (Table 3).

**TABLE 3:** Univariate and multivariate Cox regression analyses of the risk score and clinical characteristics with the overall survival in the TCGA‐LIHC cohort.

	Univariable analysis			Multivariable analysis		
Variable	HR	95% CI	*p*‐value	HR	95% CI	*p*‐value
Age (≥60 years vs. <60 years)	1.268	0.895–1.798	0.182			
Gender (male vs. female)	0.834	0.585–1.190	0.318			
Tumour stage (I/II vs. III/IV)	0.409	0.229–0.730	**0.003**	0.480	0.325–0.708	**<0.001**
Risk score (low risk vs. high risk)	0.444	0.311–0.634	**<0.001**	0.522	0.358–0.762	**<0.001**

Abbreviations: 95% CI, 95% confidence interval; HR, hazard ratio; TCGA‐LIHC, the cancer genome atlas liver hepatocellular carcinoma.

After that, the predictive abilities of the developed risk score for the 1‐, 2‐, 3‐, 4‐ and 5‐year OS were evaluated by plotting the time‐dependent ROC curves. The ROC results in GEO, TCGA‐LIHC and entire cohorts were depicted, respectively, which demonstrated acceptable predictive abilities with none of the area under curves (AUCs) that were lower than 0.600. (Figure 3D–F) Finally, principal component analysis (PCA) was performed to inspect the different distributions between the high‐ and low‐risk groups in terms of the metabolism‐related genes and global‐genome expression. The results demonstrated that the risk score developed from the metabolism‐related geneset was able to present distinctive separations both in the GEO and TCGA‐LIHC cohorts even when taking the whole‐genome expressions into consideration, indicating acceptable robustness across genesets. (Figure S3).

### 3.3 Correlation between the risk score and clinical features

**FIGURE 4:**
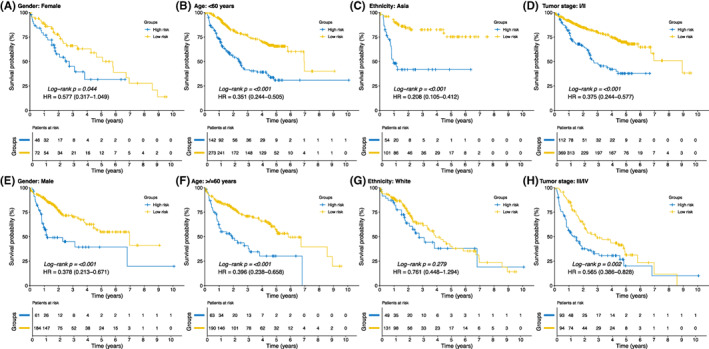
Stratified survival analysis of the high‐ and low‐risk groups in the entire cohort. (A–E) Kaplan–Meier curves for overall survival between high‐ and low‐risk groups in the patients with female and male, respectively. (B–F) Kaplan–Meier curves for overall survival between high‐ and low‐risk groups in the patients younger and older than 60 years, respectively. (C–G) Kaplan–Meier curves for overall survival between the high‐ and low‐risk groups in the patients with the ethnicity of Asia and white, respectively. (D–H) Kaplan–Meier curves for overall survival between the low‐ and high‐risk groups in the patients with I/II and III/IV stages, respectively. HR, hazard ratio.

**FIGURE 5:**
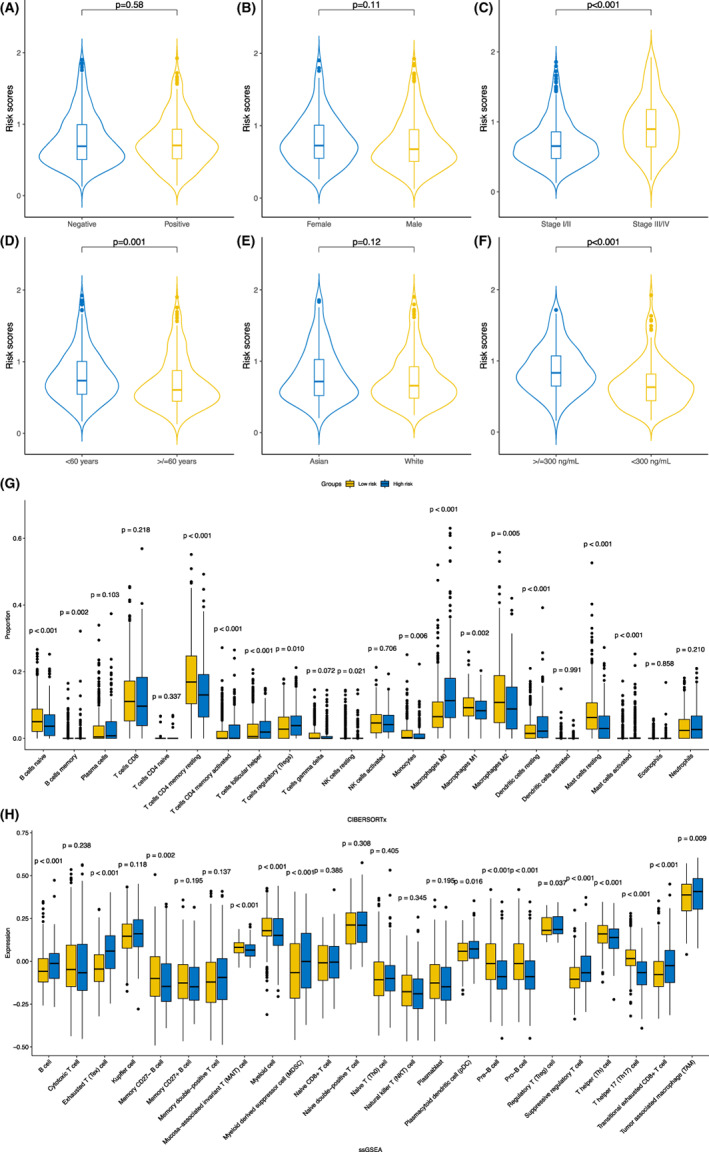
(A–F) Distributions of the risk score in different clinicopathological features in the entire cohort. (G) Comparisons of 22 infiltrated immune cells between the high‐ and low‐risk groups according to the CIBERSORTx algorithm. (H) Comparisons of sub‐population immune cells between the low‐ and high‐risk groups with the ssGSEA algorithm.

To further explore the prognostic values of the developed signature in different populations, the entire cohort was sorted into several sub‐group populations based on collected clinical features to explore K‐M curves in terms of OS between the high‐ and low‐risk groups. As results, sub‐group analyses revealed, male or female, above or below 60 years, patients from the Asia and tumour stage I/II or III/IV, the high‐risk population were significantly associated with poor prognosis. (all *p* < 0.050; Figure 4A–F,H) Interestingly, HCC patients who were race of white were unable to stratify between the high‐ and low‐risk population according to current signature. (*p* > 0.050; Figure 4G) Moreover, cases based on the entire cohort were used to explore the correlations between the risk score and clinicopathological characteristics of HCC patients. (Figure 5A–F) The result demonstrated that higher risk scores significantly corresponded to advanced tumour stage, younger age and higher AFP level. (all *p* < 0.050; Figure 5C,D,F) Whereas, no differences with respect to the risk score among hepatitis B cirus (HBV) status (*p* = 0.580; Figure 5A), different genders (*p* = 0.110; Figure 5B) and different ethnicities. (*p* = 0.120; Figure 5E).

### 3.4 Correlation between the risk score and tumour microenvironment

Subsequently, the associations between the risk score and tumour immune microenvironment were investigated via CIBERSORTx and ssGSEA algorithms. (Figure 5G,H) As for the CIBERSORTx algorithm, B cells naïve, B cells memory, T cells CD4^+^ memory resting, nature killer (NK) cells resting, monocytes, macrophages M1, macrophages M2 and mast cells resting were statistically more recruited in the samples derived from the low‐risk group. (all *p* < 0.050; Figure 5G) On the contrary, T cells CD4^+^ memory activated, T cells follicular helper, T cells regulatory (Tregs), macrophages M0, dendritic cells (DC) resting and mast cells activated demonstrated significantly more abundant density in the samples of the high‐risk group. (all *p* < 0.050; Figure 5G) Additionally, when further investigating the sub‐population of infiltrated immune cells with the ssGSEA algorithm, memory CD27^−^ B cells, mucosa‐associated invariant T (MAIT) cells, myeloid cells, pre‐B cells, pro‐B cells, T helper (Th) cells and Th17 cells were significantly more abundant in the samples classified to the low‐risk group. (all *p* < 0.050; Figure 5H) However, B cells, exhausted T (Tex) cells, myeloid‐derived suppressor cells (MDSC), plasmacytoid DC cells (pDC), Tregs, suppressive Tregs, transitional exhausted CD8^+^ T cells and tumour associated macrophages (TAMs) were statistically more enriched in the samples derived from the high‐risk group. (all *p* < 0.050; Figure 5H).

### 3.5 Validations among pan‐cancer datasets

**FIGURE 6:**
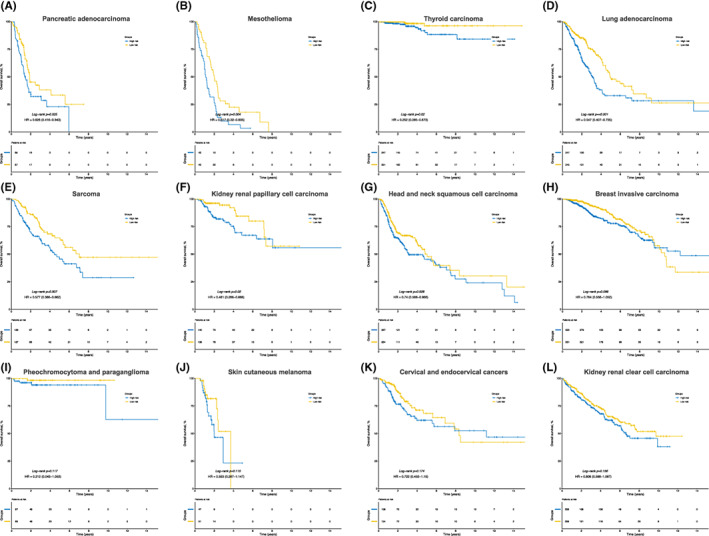
Kaplan–Meier curves for overall survival between the low‐ and high‐risk groups among the 12 types of tumour datasets from TCGA. TCGA, the cancer genome atlas; HR, hazard ratio.

To evaluate the specificity of the metabolism‐related risk score developed from HCC, the same formula was applied to the mRNA sequencing datasets of other 32 types of TCGA tumours, including 30 solid tumours and two blood system tumours. With respect to the results, the signature was able to significantly differentiate low‐ and high‐risk patients with different OS outcomes in seven types of tumours. (pancreatic adenocarcinoma, mesothelioma, thyroid carcinoma, lung adenocarcinoma, sarcoma, kidney renal papillary cell carcinoma and head and neck squamous cell carcinoma; *p* < 0.050; Figure 6A–G) Moreover, the signature was also marginally associated with OS in five types of tumours. (breast invasive carcinoma, pheochromocytoma and paraganglioma, skin cutaneous melanoma, cervical and endocervical cancers and kidney renal clear cell carcinoma; Figure 6H–L) Even though the log‐rank tests failed to reach the level of statistical significance (*p* < 0.050), the survival curves were remarkably distinct and the results of the hazard ratio were persuadable in the 5 tumours above.

### 3.6 Construction and evaluation of nomogram

**FIGURE 7:**
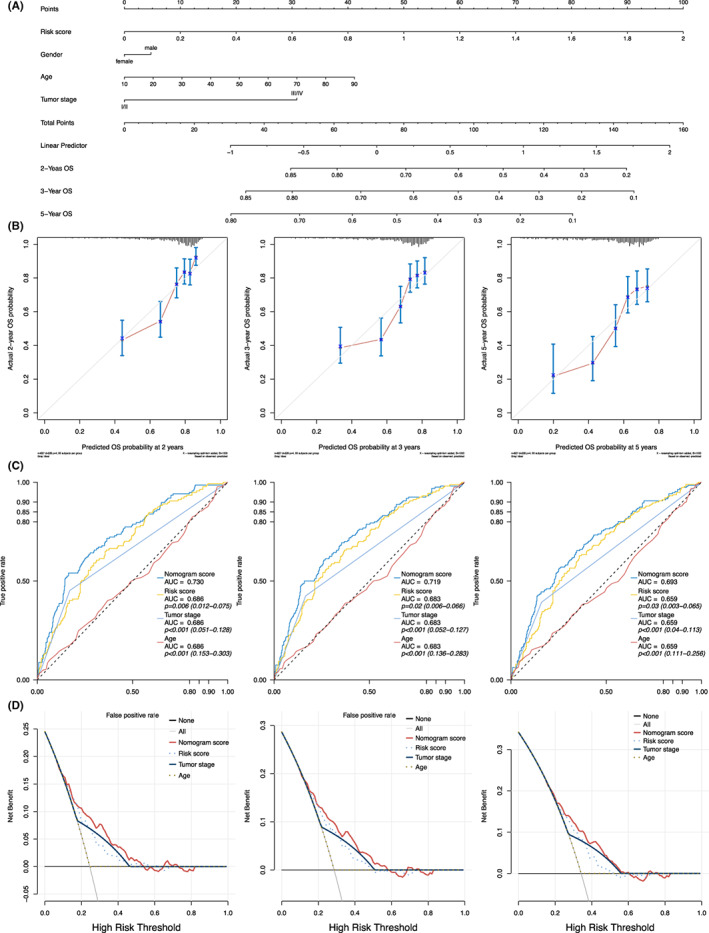
Construction and evaluation of the nomogram. (A) A nomogram that integrated the risk score, gender, age and tumour stage. (B) The calibration curves for 2‐, 3‐ and 5‐year overall survival, respectively. (C) The ROC curves for 2‐, 3‐ and 5‐year overall survival of the risk score and other clinical variables in terms of the entire cohort, respectively. (D) Decision curve analysis for the risk score, tumour stage, age and the nomogram. The solid black line represented no patients would die, and the grey line represented all patients would die. ROC, receive operating characteristic; HR, hazard ratio; AUC, area under curve; OS, overall survival.

Subsequently, to enhance the predictive ability of the current model, the developed risk score and other three clinical parameters, including gender, age, and tumour stage, were integrated into a nomogram based on the entire cohort. (Figure 7A) The 2‐, 3‐ and 5‐year OS for calibration curves of the nomogram demonstrated persuadable consistency between actual observation and predictive value. (Figure 7B) After that, the ROCs for the 2‐, 3‐ and 5‐year OS of these variables were plotted. (Figure 7C) The AUCs of the nomogram score were 0.730, 0.719 and 0.693 in terms of the entire cohorts for 2‐, 3‐ and 5‐year OS, respectively, with statistically better prognostic efficiency compared to the other variables. (*p* < 0.050; Figure 7C) Finally, DCA was conducted to compare the clinical net benefits among the nomogram, age, conventional staging system and metabolism‐related risk score. As result, the nomogram possessed improved net benefits across a wider scale of threshold probabilities for predicting 2‐, 3‐ and 5‐year OS than those of age, conventional staging system and metabolism‐related risk score. (Figure 7D).

### 3.7 Exploration of biological function

**FIGURE 8:**
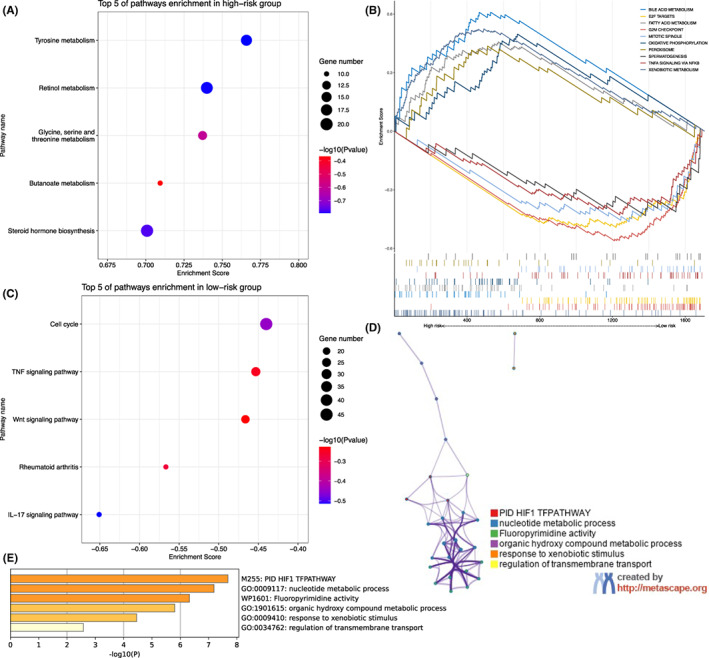
Exploration of biological function. (A–C) Bubble plots of the top five pathways enriched in the high‐ and low‐risk groups based on the Kyoto encyclopedia of genes and genomes, respectively. (B) Gene set enrichment analysis of the top five pathways significantly enriched in the high‐ and low‐risk groups, respectively. (D) The network of enriched pathways, where nodes that share the same pathway were typically close to each other. (E) Bar graph of enriched pathways across 14 metabolic genes, coloured by *p*‐values.

To explore the biological pathways underlay the signatures, first, the GSEA based on the KEGG was applied to determine the differences in terms of the hallmark pathways between the low‐ and high‐risk groups. Based on the current results, the top 5 pathways that were significantly enriched in the high‐risk groups, including ‘tyrosine metabolism’, ‘retinol metabolism’, ‘glycine, serine and threonine metabolism’, ‘butanoate metabolism’ and ‘steroid hormone biosynthesis’, were illustrated. (all *p* < 0.05; FDR, *q* < 0.25; |normalized enrichment score [NES]| ≥1; Figure 8A) And the top 5 pathways that were significantly enriched in the low‐risk groups with the same threshold, including ‘cell cycle’, ‘TNF signaling pathway’, ‘Wnt signaling pathway’, ‘rheumatoid arthritis’ and ‘IL‐17 signaling pathway’, were demonstrated. (all *p* < 0.05; FDR, *q* < 0.25; |NES| ≥1; Figure 8C) Next, the top five pathways in the high‐ and low‐risk groups referred by the hallmarks of GESA were depicted, respectively. (Figure 8B) Finally, the Metascape tool was utilized to conduct the functional annotations for the developed 14 metabolism‐related genes and to assist the determination of the underlying molecular mechanisms. (Figure 8D,E) The results revealed that the biological processes of these genes primarily engaged in the pathways nominated ‘PID HIF1 TFPATHWAY’, ‘nucleoside monophosphate biosynthetic process’, ‘Fluoropyrimidine activity’, ‘organic hydroxy compound metabolic process’, ‘response to xenobiotic stimulus’ and ‘regulation of transmembrane transport’.

## 4 DISCUSSION

Given the high heterogeneity of HCC, patients with similar tumour stages according to the TNM staging system often result in dramatically different survival outcomes, which indicated that the TNM staging system had reached its limitation in the prediction of the prognosis of HCC patients. Even though much effort was paid to explorations of optimal prognostic models utilizing molecular signatures, few biomarkers, including AFP, DCP and CA19‐9, were developed with trustworthy predictive abilities.^31^ Nevertheless, these biomarkers were mainly utilized for diagnosis or surveillance in HCC patients. With the rapid development of gene sequencing technology and an increasing understanding of tumour biological behaviours, substantial biomarkers that demonstrated prognostic values for HCC patients were identified and validated in multiple independent datasets, which promotes the accuracy of prediction for prognosis in HCC patients.^32^

For the purpose of enforcing of effectiveness and solidity of the predictive model, the present study comprehensively integrated multiple datasets (466 cases) for the construction of a novel risk score and conducted external validation based on an independent cohort with the same cut‐off value. The principal finding revealed that HCC patients stratified to high‐risk scores were negatively correlated to OS, and this observation was endorsed via the external validation of the TCGA‐LIHC cohort. With respect to the analysis of specificity for the signature, 32 types of TCGA tumours were assigned to investigate the generated 14 metabolism‐related risk score. According to the K‐M curves analysis, the risk score was associated with OS among the 12 types of tumours. One potential rationale for this phenomenon might be that the metabolism‐related signature was typically specific to several types of tumours to some extent. Among these tumours, there could be similar metabolism‐related pathways that played an essential role during tumorigenesis and progression. Illustration of these pathways would generally contribute to the research of the mechanism and development of the drug.

The current metabolism‐related risk score integrated 14 biomarkers, of which the UCK2 and PLCG2 demonstrated the maximum coefficients with aspects of pro‐ and anti‐tumorigenesis, respectively. UCK2 was reported as a typical indicator for unfavourable prognosis by regulating pyrimidine metabolism, it could also affect the immune response of HCC, which was regarded as a critical node in the treatment of HCC by most studies.^33^, ^34^, ^35^ On the other hand, PLCG2 was an unexpected biomarker found in the present study and its function was unclear until now. Given that PLCG1 recently has been proven to be a pro‐tumorigenesis gene in HCC by Seo et al., the role of PLCG2 in the procession of hepatocellular carcinogenesis was worth to be explored in the future.^36^

To validate whether gender, age, ethnicity and tumour stage could affect the predictive power of the risk score, stratification analysis was conducted to compare the OS between two groups in terms of various subgroups (male or female, older or younger than 60 years, ethnicity of Asia or White and tumour stage I/II or III/IV). The results revealed that OS advantages for the low‐risk group could be observed in either all genders, age groups or different tumour stage subgroups, indicating that the risk score has a broad utility among HCC patients. Interestingly, when stratified patients according to ethnicity, OS benefits of low‐risk HCC patients could only be addressed with patients from the Asian area. One possible reason for these results might be that the HCC in the Asian were more commonly developed from the chronic hepatitis B/C viral background, which indicated that the present metabolism‐related risk score were more sensitive towards HCC patients who possessed this characteristics.^37^, ^38^ Additionally, as integrated metabolism‐related risk score, age, gender and TNM stage into a nomogram, nomogram scores for each patient were able to be calculated. Based on the results of 2‐, 3‐ and 5‐year ROCs and DCA, the developed nomogram demonstrated superior predictive ability to that of the conventional staging system (all *p* < 0.050), suggesting that the metabolism‐related risk score combined with other clinical information was able to achieve a robust prediction for prognosis. The clinicians would benefit from genetic detection to obtain information of expressions for the 14 metabolic genes in the future clinical practice. Via integrating clinical and genetic parameters mentioned above, the current nomogram could precisely calculate the specific OS probability of each patient, which further promoted the individualized clinical decision making of HCC patients.

Substantial studies reported that the immune cell infiltration in the tumour microenvironment (TME) was of clinical importance for surveillance and treatment.^39^, ^40^, ^41^ Recently, Liu et al. reported that infiltrating immune cells was an independent prognostic biomarker in early‐stage HCC, and estimated its value in predicting short‐term outcomes after hepatectomy.^42^ Accordingly, in the present study, the differential abundance of tumour‐infiltrating immune cells between the low‐ and high‐risk groups were compared according to the CIBERSORTx and ssGSEA algorithms, respectively. The CIBERSORTx algorithm discovered 14 types of immune cells which showed significantly different distributions between the two groups. Among them, B cells naïve, B cells memory and M1 phenotype macrophages were more recruited in the low‐risk group. Zhang et al. reported that high densities of tumour‐infiltrating B cells implied better clinical outcomes in HCC.^43^ Moreover, an extensive literature has indicated that tumour‐infiltrating B cells facilitate to a positive prognostic effect for cancer.^44^ Zhang et al. indicated that infiltration of M1 phenotype macrophage was able to inhibit HCC metastasis.^45^ Furthermore, when exploring the subsets of tumour‐infiltrating immune cells with the ssGSEA algorithm, the Tex cells, MDSCs, transitional exhausted CD8^+^ T cells, suppressive Tregs and TAMs were more enriched in the high‐risk group. Plenty of evidence revealed that myeloid cells including the MDSCs and TAMs were associated with poor prognosis for HCC.^46^ And Barsch et al. reported that the enrichment of Tex cells in the TME represented poor survival compared to patients with a predominance of tissue‐resident memory T cells in HCC tissue.^47^ Therefore, the above tumour‐infiltrating immune cell patterns might support the explanation of the outcomes that the low‐risk group had a better prognosis compared to the high‐risk group in HCC patients.

Additionally, the biological processes with respect to the low‐ and high‐risk groups were investigated via KEGG and GSEA analysis. The results indicated that a total of 5 pathways were significantly enriched in the high‐risk group, of which the ‘tyrosine metabolism’ pathway was regarded as critical cyclins and cyclin‐dependent kinases in the procession of tumorigenesis.^48^ Accordingly, molecularly targeted drugs, including sorafenib and lenvatinib, have been developed to block this pathway and were utilized as first‐line therapy for advanced HCC.^49^, ^50^ Moreover, the ‘IL‐17 signaling pathway’ was enriched in the low‐risk group mostly and it revealed that the inflammation played a vital role in the tumorigenesis in liver tissue from cirrhosis to HCC.^51^ Ma et al. reported that IL‐17A was able to critically regulate inflammatory responses in the kupffer cells, bone‐marrow‐derived monocytes and cholesterol synthesis in steatotic hepatocytes in an experimental model of alcohol‐induced HCC, which might be a potential therapeutic target for alcohol‐induced HCC patients.^52^ Taken together, these enriched pathways were predominantly associated with hepatocellular tumorigenesis and metabolic alterations. Exploring the underlying molecular mechanisms helps in the development of new therapeutic targets for HCC.

Whereas, the current study suffered from several limitations. First, the enrolled research datasets were derived from the public database, which caused it difficult to collect complete clinical information for each patient. Regardless, since this study involved multiple series of sequence datasets, the batch effect was inevitable despite applying the LASSO‐Cox and LASSO‐pcvl algorithms to eliminate it. Therefore, the results should be interpreted with caution. Last but not least, given the retrospectively designed, the potential bias correlated with unbalanced clinicopathological profiles was unable to be ignored. Prospective, large‐scale cohort were urgently required to validate the prognostic value of the present developed metabolism‐related gene signature.

## 5 CONCLUSION

In the present study, for the first time, a list of metabolic genes related to OS was identified and developed a metabolism‐related risk score for HCC patients. Undergoing a series of bioinformatics and statistical analyses, the predictive ability of the signature was approved. It was believed that the current signature would induce the discovery of a novel landscape for the therapeutic strategy of the HCC in the future.

## AUTHOR CONTRIBUTIONS

**Yang‐Xun Pan:** Conceptualization (lead); data curation (lead); resources (lead); software (lead); writing – original draft (lead). **Deyao Zhang:** Conceptualization (equal); resources (lead); software (equal); validation (equal). **Yuheng Chen:** Investigation (equal); methodology (equal); software (lead). **Huake Li:** Project administration (equal); visualization (equal). **Jiongliang Wang:** Formal analysis (equal); software (equal). **Ze Yuan:** Formal analysis (equal); validation (equal). **Liyang Sun:** Methodology (equal). **Zhong‐Guo Zhou:** Validation (equal). **Min‐Shan Chen:** Supervision (lead); validation (equal); writing – review and editing (equal). **Yao‐Jun Zhang:** Formal analysis (equal); supervision (equal); validation (equal); visualization (supporting). **Dandan Hu:** Writing – review and editing (lead).

## FUNDING INFORMATION

This work was funded by National Natural Science Foundation of China (82103566).

## CONFLICT OF INTEREST STATEMENT

The authors have no conflict of interest to declare.

## Supporting information


Figure S1.

Figure S2.

Figure S3.

## Data Availability

The datasets generated for this study can be found in the GEO database (GSE14520, GSE54236, GSE10141 and GSE116174; https://www.ncbi.nlm.nih.gov/geo/), and UCSC Xena website (https://gdc.xenahubs.net).
