# Supplementary figures and images for: Development and validation of robust metabolism‐related gene signature in the prognostic prediction of hepatocellular carcinoma
Source: J Cell Mol Med. 2023 Mar 15;27(7):1006–20. doi: 10.1111/jcmm.17718 (PMC10064027; doi:10.1111/jcmm.17718)

**A** Before batch effect correction

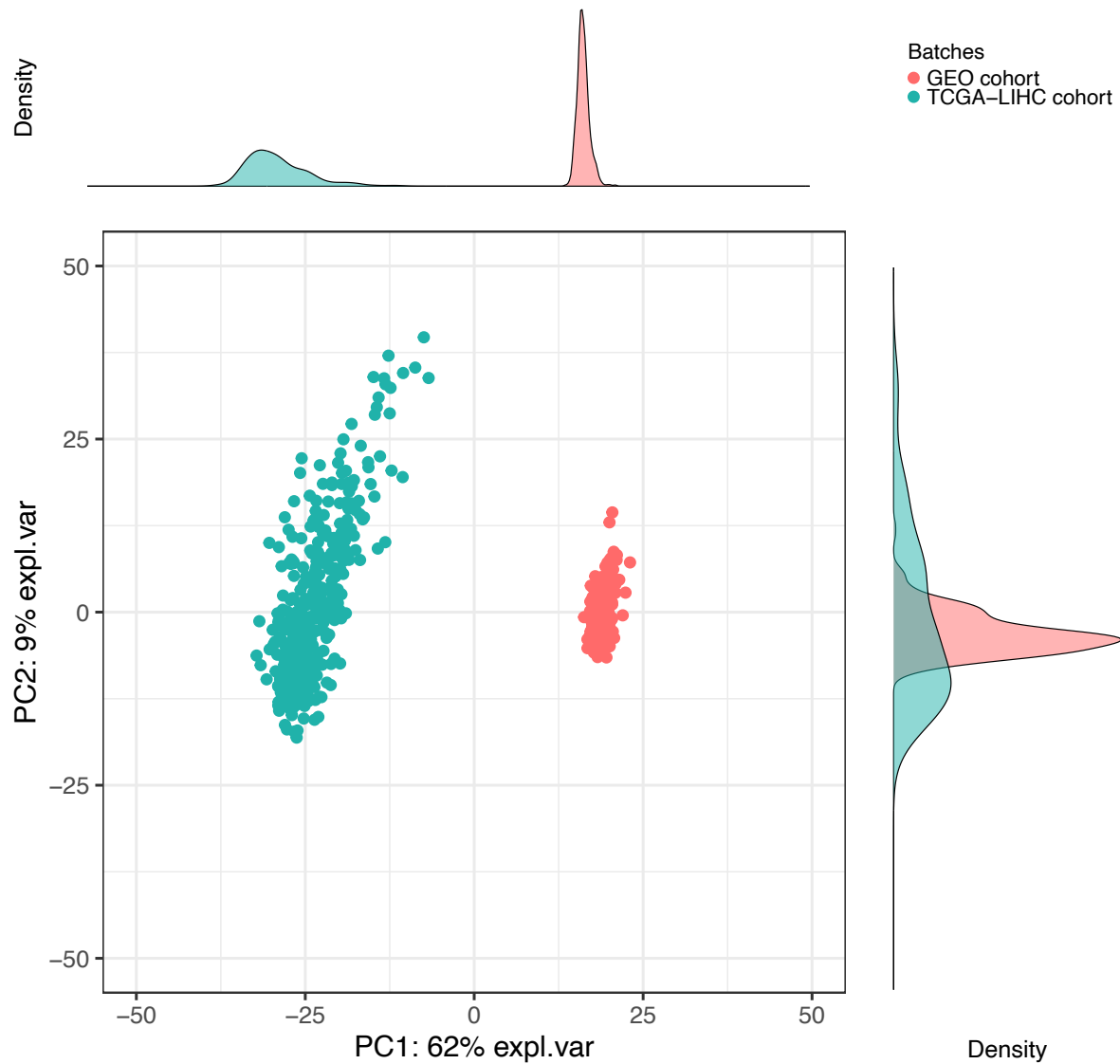

**B** After batch effect correction

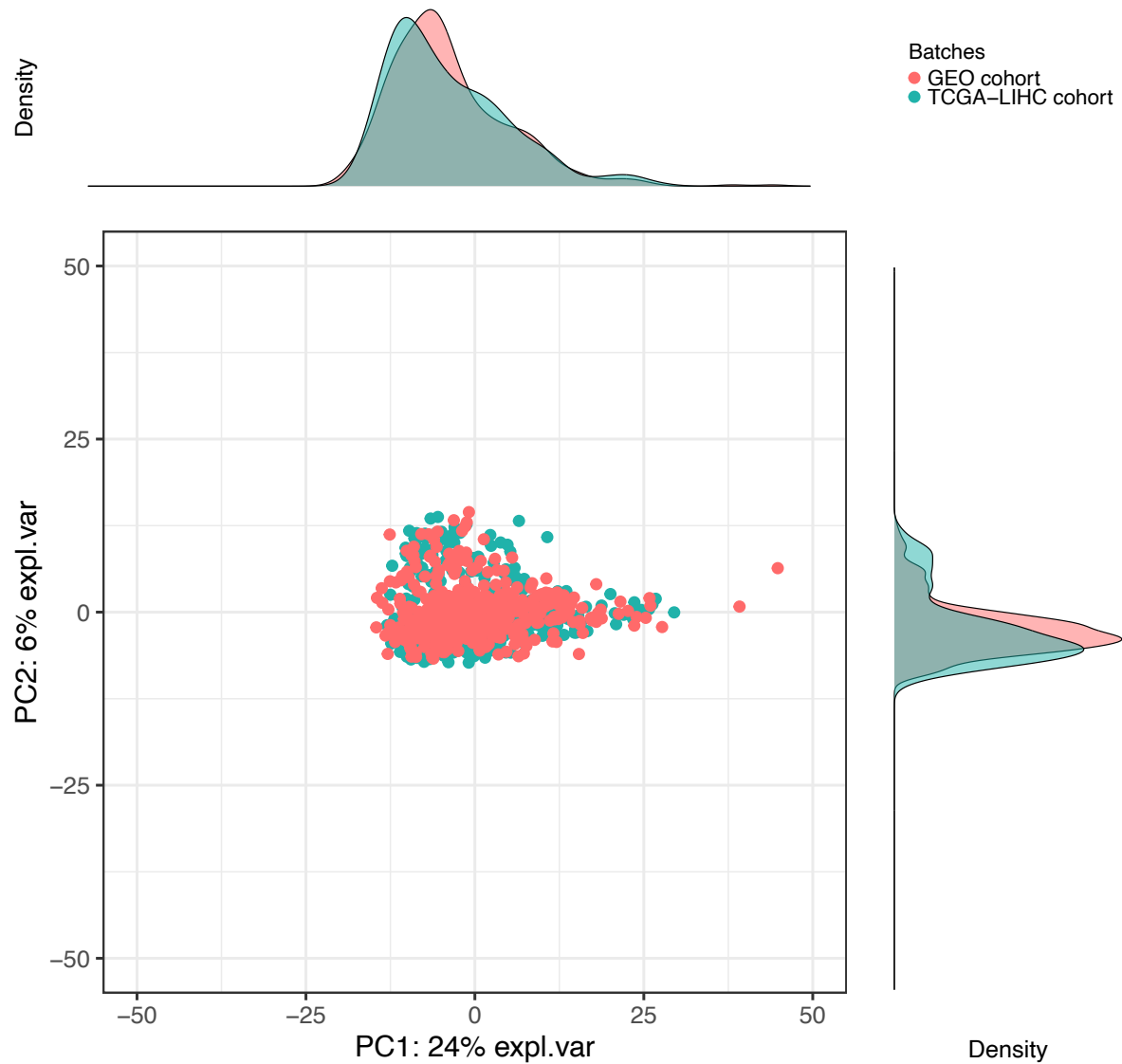

Supplement: Supplementary file 1 — Figure S1. [file JCMM-27-1006-s003.pdf]

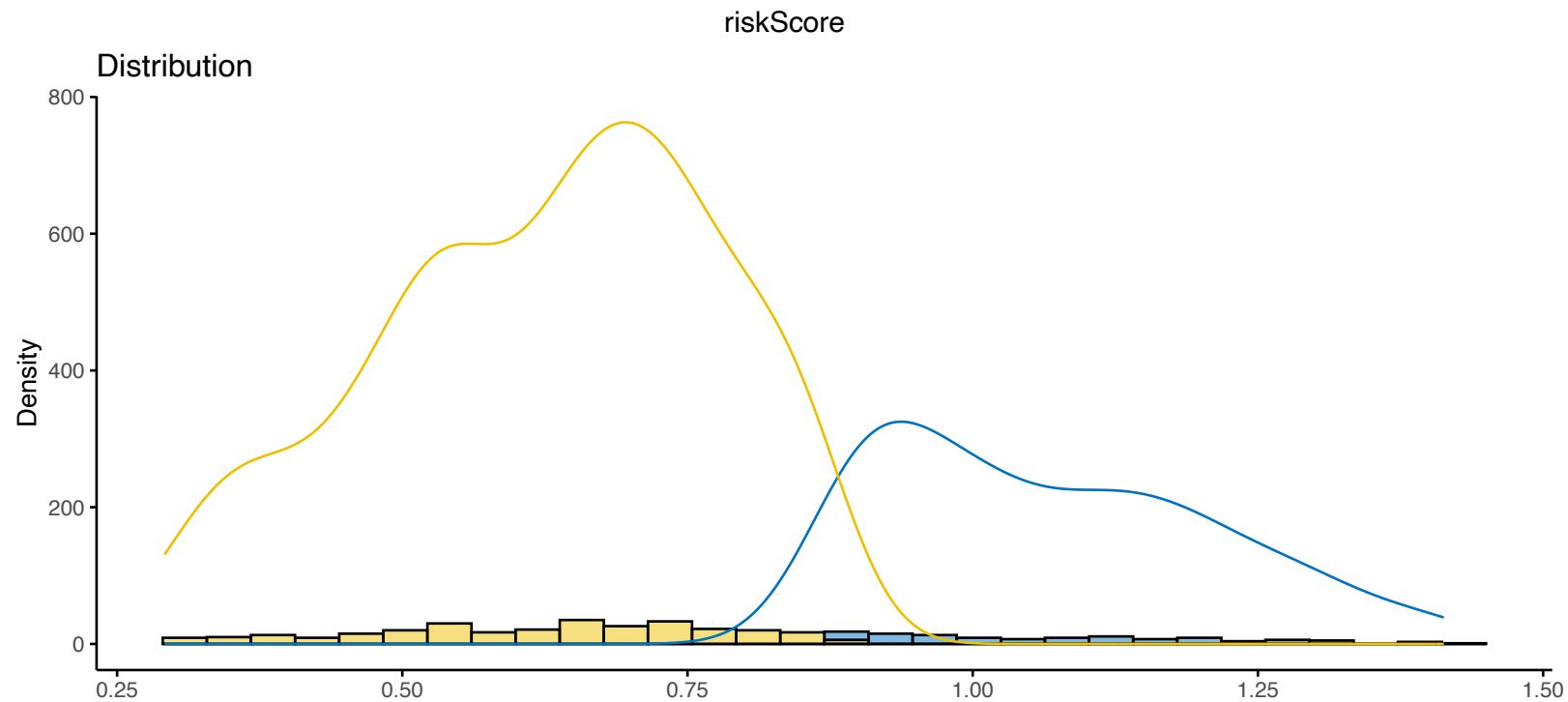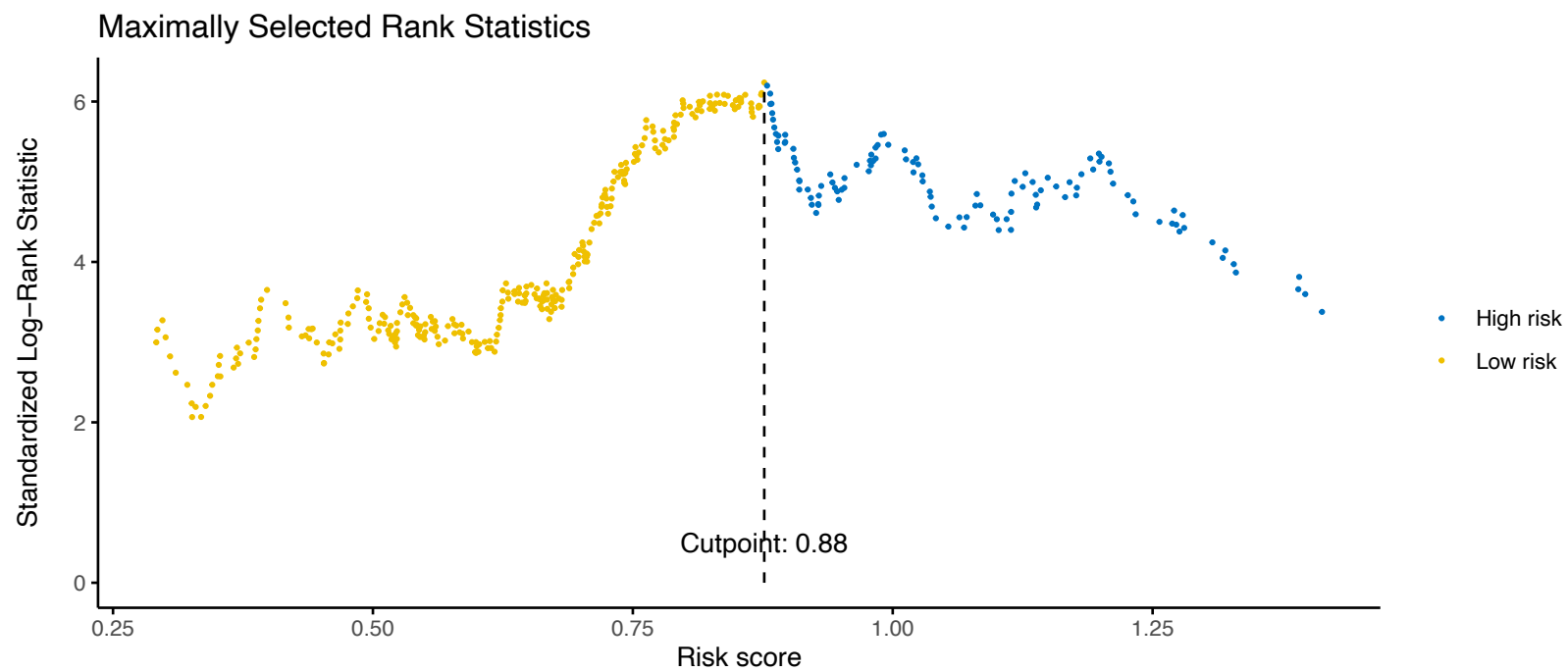

Supplement: Supplementary file 2 — Figure S2. [file JCMM-27-1006-s001.pdf]

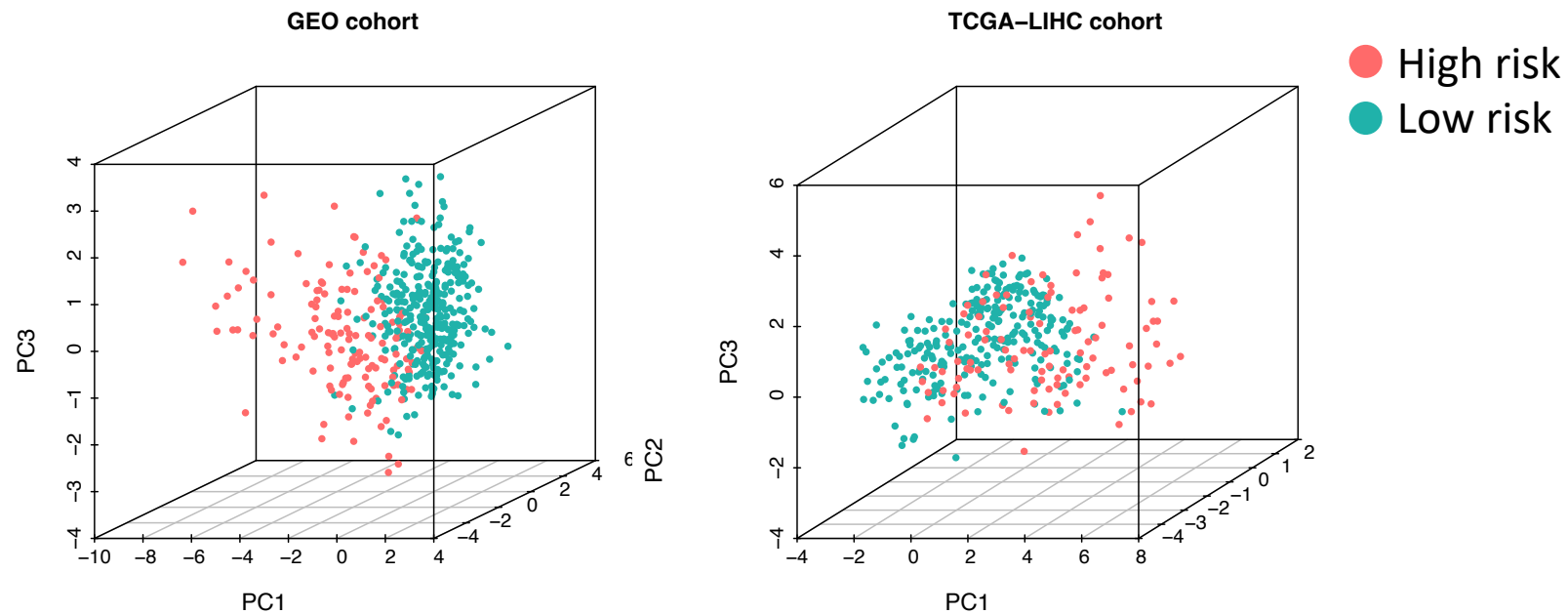

**(A) Metabolism-related gene sets**

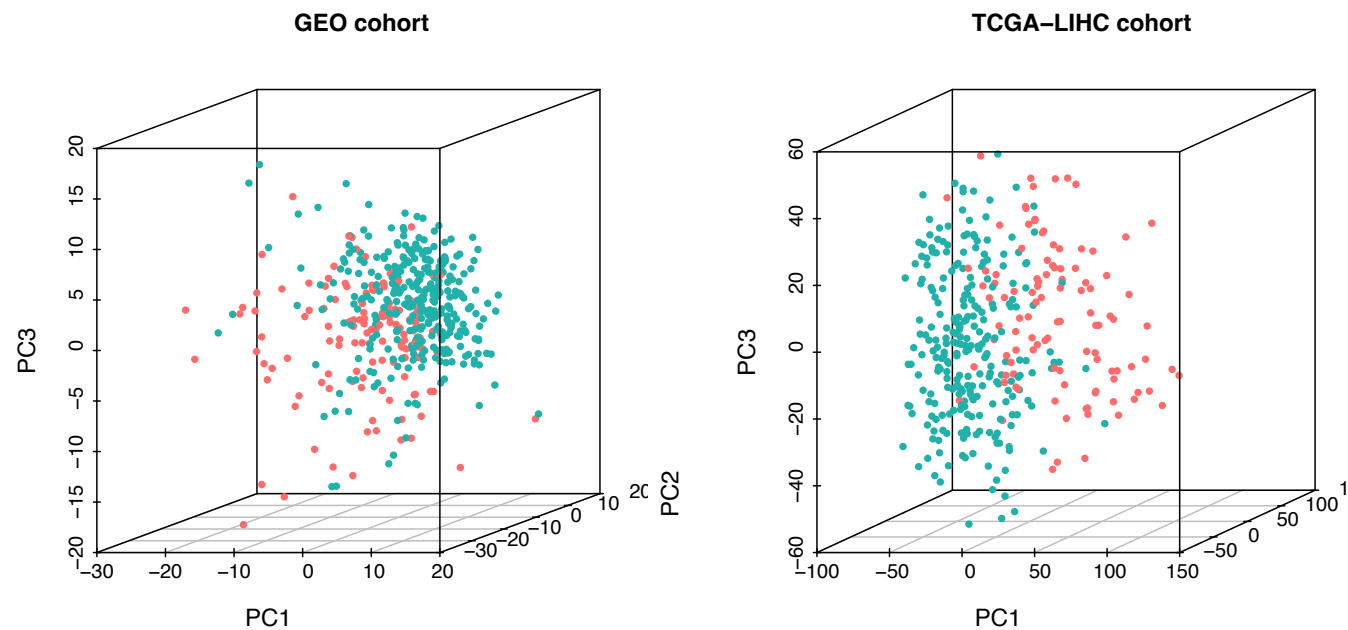

**(B) Global gene sets**

Supplement: Supplementary file 3 — Figure S3. [file JCMM-27-1006-s002.pdf]
